# Woolly apple aphid *Eriosoma lanigerum* Hausmann ecology and its relationship with climatic variables and natural enemies in Mediterranean areas

**DOI:** 10.1017/S0007485314000753

**Published:** 2014-10-22

**Authors:** Jaume Lordan, Simó Alegre, Ferran Gatius, M. José Sarasúa, Georgina Alins

**Affiliations:** IRTA – Fruitcentre, Parc Científic i Tecnològic Agroalimentari de Lleida. Parc de Gardeny, edifici Fruitcentre, 25003 Lleida, Spain

**Keywords:** *Aphelinus mali*, crawler, European earwig, *Forficula auricularia*, multivariate analysis, winter survival

## Abstract

A multilateral approach that includes both biotic and climatic data was developed to detect the main variables that affect the ecology and population dynamics of woolly apple aphid *Eriosoma lanigerum* (Hausmann). Crawlers migrated up and down the trunk mainly from spring to autumn and horizontal migration through the canopy was observed from May to August. Winter temperatures did not kill the canopy colonies, and both canopy and root colonies are the source of reinfestations in Mediterranean areas. Thus, control measures should simultaneously address roots and canopy. European earwigs *Forficula auricularia* (Linnaeus) were found to reduce the survival of overwintering canopy colonies up to June, and this can allow their later control by the parasitoid *Aphelinus mali* (Haldeman) from summer to fall. Preliminary models to predict canopy infestations were developed.

## Introduction

Woolly apple aphid (WAA), *Eriosoma lanigerum* (Hausmann) (Hemiptera: Aphididae), is a worldwide pest of apple *Malus domestica* (Borkhausen). It is a native of North America, where the American elm *Ulmus americana* (Linnaeus) (Urticales: Ulmaceae) is the primary host and apple the secondary one; in the absence of the primary host it develops on apple throughout the year.

The biology of WAA has been widely studied in the USA (Hoyt & Madsen, 1960; Walker, 1985; Walker *et al*., 1988; Brown & Schmitt, 1994; Beers *et al*., 2007,  2010), New Zealand (Alspach & Bus, 1999; Sandanayaka & Bus, 2005), Australia (Asante *et al.*, 1993; Asante, 1994, 1999) and South Africa (Pringle & Heunis, 2001, 2008; Heunis & Pringle, 2006; Damavandian & Pringle, 2007;). However, little information is available in Europe (Theobald, 1921; Evenhuis, 1958), especially in Mediterranean areas.

This aphid colonizes roots and sites on the trunk and branches that have been previously injured, and can also colonize undamaged current year shoots (Childs, 1929; Weber & Brown, 1988; Brown *et al.*, 1991; Asante *et al.*, 1993; Asante, 1994; Pringle & Heunis, 2001; Beers *et al.*, 2010). WAA is distributed irregularly across the orchard, gathering on given trees or along isolated rows (Asante *et al.*, 1993). The principal dispersion method between trees involves first instar nymphs (crawlers), which are transported by orchard management practices, migration or wind (Schoene & Underhill, 1935; Nel, 1983; Walker, 1985).

Several studies have linked canopy infestations with the upward movement of crawlers from the roots, suggesting that the root colonies are the constant source of canopy infestations (Theobald, 1921; Nel, 1983; Heunis & Pringle, 2006). This can be especially important in areas where canopy colonies are highly affected by low winter temperatures (Walker, 1985), but the role that these cold temperatures may have on canopy colonies in Mediterranean areas has not been checked.

The increase in WAA outbreaks appears to be associated with changes in pesticide programs and the disruption of biological control (Gontijo *et al.*, 2012). Information on the efficacy of WAA parasitoid *Aphelinus mali* (Haldeman) (Hymenoptera: Aphelinidae) to control arboreal populations is contradictory. Therefore, while in warmer regions, such as Brazil, no chemical control is necessary due to high parasitism rates (Monteiro *et al.*, 2004), under cool climatic conditions *A. mali* is not effective in preventing economic damage (Asante & Danthanarayana, 1992; Heunis & Pringle, 2006). Predators such as ladybird beetles (Coleoptera: Coccinellidae), lacewings (Neuroptera: Chrysopidae), hoverflies (Diptera: Syrphidae), earwigs (Dermaptera: Forficulidae) and spiders (Araneae) are reported to be WAA predators; of these, earwigs are cited as the most important (Mueller *et al.*, 1988; Asante, 1995, 1997; Short & Bergh, 2004; Nicholas *et al.*, 2005; Gontijo *et al.*, 2012). However, very few data are available on the efficacy of earwigs to control WAA in the Mediterranean area.

Aims of this study were to know the ecology of WAA in Mediterranean areas, the winter survival of the canopy colonies and the role that natural enemies may play in such areas in order to improve WAA control. A multilateral approach that includes both biotic and climatic data was developed to detect the main variables that affect WAA ecology and population dynamics.

## Materials and methods

### Study orchards

Trials were performed in three apple orchards located in Catalonia (NE Spain): les Borges Blanques (BB) (41°30′23.06″N; 0°51′05.93″E), Mollerussa (MO) (41°36′51.13″N; 0°52′22.75″E) and Ivars d'Urgell (IU) (41°41′06.19″N; 0°58′06.09″E). The climate is semi-arid Mediterranean, with a mean annual rainfall of 350 mm. All the orchards had major infestations of WAA and were under organic management. The orchards were treated with pesticides as follows: Azadirachtin, maximum twice a year around the end of March–April to control rosy apple aphid (*Dysaphis plantaginea* (Passerini), Hemiptera: Aphididae), before WAA aerial infestations (AIs) initiate their development; granulosis virus in April and May against codling moth (*Cydia pomonella* (L.), Lepidoptera: Tortricidae); and lime sulfur from April to May to control apple scab (*Venturia inaequalis* Cooke). In addition, to control codling moth, Spinosad was applied twice to IU in June and July 2012.

BB was an IRTA (Institute of Research and Technology, Food and Agriculture) experimental orchard of ‘Fuji Kiku 8’ apple grafted onto M9, planted in 2003, and trained to a central leader with a spacing of 4×1.4 m. MO was a commercial orchard of ‘Golden Smoothee’ apple grafted onto M9, planted in 1985, and trained to a double-axis system with a spacing of 4×1.2 m. IU was a commercial orchard of ‘Golden Smoothee’ apple grafted onto M9, planted in 1993, and trained to a central leader with a spacing of 4×1.1 m. BB and MO were drip-irrigated, whereas IU was flood-irrigated.

Hourly climatic variables such as maximum temperature (Tmax, °C), minimum temperature (Tmin, °C), number of hours above or below several temperature thresholds (h>20 °C,  25 °C, <10 °C and <7 °C), minimum relative humidity (rh min%), solar radiation (Sun, W m^−2^), rainfall (Rain, mm) and wind speed (Wind, m s^−1^), were obtained from the closest automatic weather station of the Meteorological Service of Catalonia (*Meteocat, Departament de Territori i Sostenibilitat, Generalitat de Catalunya*). For BB, data were obtained from the Castelldans station 8.5 km away, for IU from the Castellnou de Seana station 3 km away and for MO from the Mollerussa station 0.5 km away.

### Crawler movement

To assess crawler movement from root and aerial colonies, 50 trees with WAA infestations were selected in each orchard. BB was sampled for 3 years (2010–2012), whereas MO and IU were sampled for two (2011–2012).

Upward (from root colonies) (Up) and downward (from aerial parts) (Down) crawler movement was evaluated weekly in 20 trees over the whole year. Of these trees, 10 were consistently included in the evaluation, whereas the other 10 rotated every week, being repeated every 4 weeks in order to minimize interference with WAA phenology.

For each tree, two 2.5-cm-wide adhesive tapes (Tesa Tape S.A.; Argentona, Spain) placed 3 cm apart were wrapped around the trunk above the graft union. A thin bead (1.5-cm-wide) of insect trapping medium (Tree Tanglefoot; the Tanglefoot Company, Grand Rapids, MI) was centered along each tape. Aphids moving up from the root colonies were trapped on the lower tape, while those moving down from the canopy were trapped on the higher one. Tapes were replaced weekly throughout the year, and WAA number on each tape was visually estimated by a qualitative index of six categories. This index was developed through a geometrical scale (*a*_*n*_=*a*·*r^n^*^−1^) where *r*=3, *a*=4 and *n* is from 2 to 7 (table 1). The use of this scale allowed us to adopt the same index category regardless of trunk diameter. For data analysis, categories were transformed to the mean aphid number of each interval (table 1).

**Table 1:** Interval and mean number of aphids for each category according to the qualitative index.

Category	Number of aphids	Mean
1	0–12	6
2	13–36	25
3	37–108	73
4	109–324	217
5	325–972	649
6	973–2916	1945

In addition, the numbers of *A. mali* and the most abundant predators, such as spiders, earwigs and velvet mites (Trombidiformes: Trombidiidae), trapped on each tape were recorded as an indicator of presence. Given that earwigs are considered the most important predator of WAA and we were unsure whether the tapes would trap them, their number was also assessed by means of shelters. For this purpose, we set up 10 earwig shelters on the second scaffold limb of 10 different trees randomly selected within the infested ones in each orchard. According to Lordan *et al.* (2014), the shelters were prepared by rolling a piece of corrugated cardboard into a cylinder (12 cm height×9 cm diameter), which was protected from rain and adverse conditions by a PVC tube (15 cm height×9.5 cm diameter). Similar shelters have been used in studies of European earwigs elsewhere (Phillips, 1981; Helsen *et al.*, 1998; Solomon *et al.*, 1999; Burnip *et al.*, 2002; Gobin *et al.*, 2006; Logan *et al.*, 2007; He *et al.*, 2008; Moerkens *et al.*, 2009). Every week throughout the year, we counted the number of earwigs per shelter. After counts, the insects were released at the base of the assessed tree.

Horizontal movement between trees through the canopy (C) was assessed fortnightly from May to December 2012. In each orchard, 10 of the trees used to assess the crawler movement were included. Five of these were permanently taped, while the other five were those taped every 4 weeks. One glue tape (described above) per tree was wrapped around a branch that was in contact with branches of a neighbor tree; these branches were randomly selected for every assessment. The tapes were removed 1 week later and aphids were individually counted under a stereomicroscope.

### WAA AI and parasitism

This study was carried out from May through December for 2 years (2011–2012). To assess the canopy infestation, 20 trees per orchard were used. Ten trees with permanent trunk tapes used to evaluate crawler movement (section above) were included, together with another 10 WAA-infested trees that had never been trunk-taped. For each tree, five shoots were randomly selected. Every 2 weeks, the total length of the shoot and the length occupied by WAA were measured to calculate the percentage of the AI. The percentage of infested shoots (IS) was evaluated at the same time. Also, the percentage of the length of each colony parasitized by *A. mali* (parasitism) was assessed visually using a qualitative scale (<10, 10–50, 51–90% and >90%). The mean value for each category was used to represent and analyze parasitism. In each orchard, the same 20 WAA-infested trees were used during the 2 years of evaluation.

### Winter survival of WAA aerial colonies

This study was carried out in the BB orchard in 2012. At the beginning of February, the coldest month in our area, 75 shoots that had similar levels of WAA infestations the previous summer were selected. Of these, 25 were covered with a cloth bag to exclude natural enemies and WAA recolonization, 25 were glue-taped (trapping medium) at the base to prevent WAA recolonization, and the other 25 were used as controls. The glue was checked regularly to ensure its effectiveness. At the end of June, when aerial colonies reach their maximum development, AI was evaluated. The air temperature inside and outside the cloth bag was recorded by data loggers (Testo 177-T4; Testo AG; Lenzkirch, Germany) over 3 weeks in February. For this purpose, five control shoots and five shoots covered by a cloth bag were randomly selected, and a temperature sensor was placed on each one.

### Data analysis

The annual cumulative number of aphids captured moving up and down was analyzed per year within orchards by one-way ANOVA; data were log-transformed and ANOVA assumptions (normality and homoscedasticity) were confirmed before analysis. Tukey-HSD tests were used to compare means. The number of aphids captured on trees that were permanently taped and trees that were included in the evaluation every 4 weeks was log-transformed and analyzed by a non-parametric Wilcoxon test. To evaluate AI at the end of the winter survival trial, data were tested for significance by a non-parametric Kruskal–Wallis test, and the Steel–Dwass method was used to separate treatments. These non-parametric tests were used because the ANOVA assumptions were violated. Temperature inside and outside the shoot bags was analyzed by one-way ANOVA. Data were analyzed using the JMP statistical software package (Version 9; SAS Institute Inc., Cary, North Carolina).

Multivariate projection methods were applied to simultaneously analyze biotic and abiotic variables. For this purpose, we used the following variables for each orchard and year: the weekly number of aphids captured on the bands (Up, Down and C), the accumulated number of aphids captured each week (Up ac and Down ac), the AI, IS, the mean values of the classes of parasitism, the number of earwigs and *A. mali* individuals captured on the bands (EarwC and MaliC, respectively), and the number of earwigs present in shelters (EarwP). For every week that crawler movement and AI were evaluated, a mean value of each climatic variable was calculated, with the exception of rainfall, for which accumulated rain was used. All the variables were analyzed in the same matrix.

We performed a PCA and a regression model by PLS for one-dependent variable (PLS-1) and two dependent variables (PLS-2). Regression procedures by means of PLS-1 methods were carried out to predict the Up and Up ac variables, whereas the AI and IS variables were studied together by means of a PLS-2 technique. According to their contribution to explain the overall variance in the PCA and to the easiness to obtain them, the X-variables used to construct the PLS-1 were: MaliC, Parasitism, Tmax, Tmin, Wind, Sun, Up, EarwP, rh min%, Rain, h <7 °C and h <10 °C. To construct the PLS-2, the X-variables used were: AI, IS, Parasitism, Up ac, Tmax, Tmin, Wind, Sun, EarwP, rh min%, h <7 °C and h <10 °C. Before analysis, all the data were centered and standardized by dividing each variable by its standard deviation. Both the PCA and PLS models were validated using the full cross-validation method. All these multivariate models were performed using The Unscrambler software (Version 7.6; Camo Process AS, Oslo, Norway).

## Results and discussion

### Within-tree WAA crawler movement in Mediterranean areas

For all the orchards and years, no differences were observed between trees that were taped every 4 weeks and those taped continuously (data not shown). Therefore, data were pooled for the analysis.

**Fig 1.**
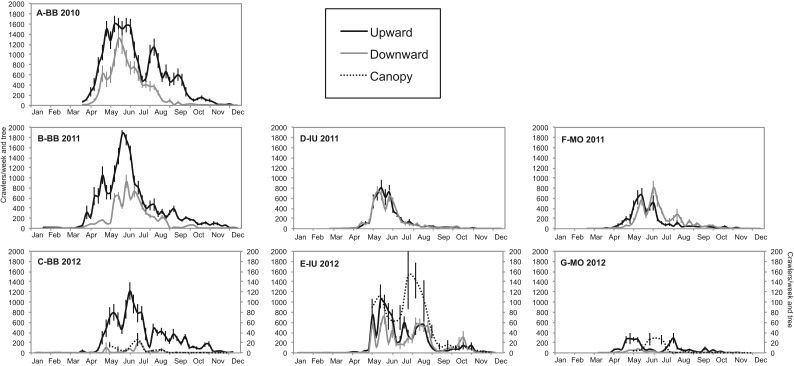
Number of woolly apple aphid crawlers captured per tree per week (mean±SEM) throughout the year. Note that crawlers through the canopy are referred to the secondary axis and are present only in 2012.

Crawler movement was recorded almost year-round in all the orchards, although with very low number of crawler catches from fall to early spring (fig 1). Peak captures were observed from May to June, and in some years and orchards there seemed to be two annual peaks (fig 1), probably due to fluctuation of the maximum temperatures in summer. These relations are addressed more in detail in the multilateral approach analysis. The up:down ratio of accumulated crawlers was highly variable even in the same orchard (fig 1 and table 2). We observed ratios from 1:1 (IU both years) to 11:1 (BB 2012) (fig 1 and table 2).

**Table 2.** Number (mean±SEM) of annual cumulative woolly apple aphid crawlers per orchard on the lower (Up) and upper (Down) bands.

Orchard/year	Up accumulated	Down accumulated
BB		
2010	23,684±2257a	10,885±1417a
2011	18,867±1055a	7380±553a
2012	12,646±1094b	1097±146b
d.f.	2,57	2,57
*F* value	7.63	51.33
Prob>*F*	0.0012	<0.0001
IU		
2011	5375±910	5145±795
2012	9377±1433*	7228±1,492
d.f.	1,38	1,38
*F* value	7.88	1.12
Prob>*F*	0.0078	ns
MO		
2011	4897±704*	5504±807*
2012	2814±373	656±44
d.f.	1,38	1,38
*F* value	5.72	126.25
Prob>*F*	0.0218	<0.0001

Column values followed by different letters or asterisk indicate significant differences within orchards, as determined by the Tukey-HSD test (*P*<0.05).

Although it is difficult to extrapolate the results of three orchards to the whole area, some common aspects can be highlighted. For example, the captures on the trunk tapes, which show the pattern of upward and downward crawler movement, occurred consistently from mid-April to November with a plateau around May–June, while the movement across the canopy was higher from May to August (fig 1). The maximum number of aphids captured per tree over 1 week (1800 upward captures) occurred in BB in 2011 (fig 1B). Analogous results, using similar sampling methods, were found by Beers *et al.* (2010) in Washington, where crawler movement started in May but diminished considerably after July, and the migration pattern resembled a peak rather than a plateau, with a maximum of 1500 upward crawlers per tree per week. In California, with a similar Mediterranean climate, Hoyt & Madsen (1960) observed year-round crawler movement and, despite increasing in May and June, the highest level was observed in July and August, declining from September onwards. A year-round migration pattern with peaks in late spring and from late summer to autumn was also reported by Asante (1994) in Australia and by Heunis & Pringle (2006) in South Africa, with the greatest movement occurring from October to December (equivalent to April–June in the Northern hemisphere).

Regarding the captures of crawlers moving through the canopy, the highest captures were from June onwards, following the same pattern as the captures of downward crawlers, and immediately after the peak of upward movement was recorded (fig 1). Asante *et al.* (1993) observed that at low infestations the aphid is confined to the trunk and large branches, but disperses to establish colonies on twigs or new lateral growths during peak populations. Taking into account only the movement of crawlers, we could not find a consistent relationship between canopy and root colonies. The same observation was made by Beers *et al.* (2010) in Washington. Therefore, to detect the main driving variables that explain the dynamics of WAA, a multilateral approach that includes both biotic and climatic data would be more appropriate than trying to separate the contribution of each individual factor.

### WAA winter survival and role of natural enemies

In our study, low winter temperatures did not kill aerial colonies of WAA. High AI rates were observed on shoots on which recolonization by crawlers and access of natural enemies were prevented by cloth bags (table 3). Shoot temperature was only 0.7 °C higher in bag-covered shoots than in control ones (*F*=23.8011; d.f.=1,10606; *P*<0.0001), and as no differences in AI were observed between shoots without bags (glue and control) and those with bags containing earwigs, bag protection against cold was discarded. Therefore, the effect of subterranean WAA populations on AI is expected to be less significant than in areas where aerial colonies are killed or reduced, for instance in central Washington, where Walker (1985) observed high mortality in winter, or in South Africa, where Heunis & Pringle (2006) stated that AIs originate every year from the roots.

**Table 3.** Aerial infestation (AI; percentage of shoot length occupied by woolly apple aphid, mean±SEM) at the end of June 2012 for the BB orchard in the winter survival trial.

Treatment	AI (%)
Bag (*N*=9)	59.2±8.5a
Bag with earwigs (*N*=16)	10.0±4.0b
Glue (*N*=25)	5.9±2.0b
Control (*N*=25)	2.7±0.8b
d.f.	3
χ^2^	25.89
Prob>χ^2^	<0.0001

Values followed by different letters indicate significant differences, as determined by the Kruskal–Wallis test and Steel–Dwass method (*P*<0.05).

Although no significant differences were observed among control, glue and bags with earwigs, there was a trend suggesting that the less isolated the shoots were, the less AI was found. This observation could be attributed to the difficulty encountered by predators to reach them. Earwigs had entered some of the bags used to assess winter survival (16 of the initial 25) through small holes, probably made by the earwigs themselves. AI was close to 60% on bag-isolated shoots (the remaining nine) and reached only 10% on shoots with earwigs (table 3). The glue at the base of some shoots prevented crawler recolonization, but it was not enough to impede the movement of earwigs. Thus, earwig exclusion on shoots with glue was also discarded. This observation in addition with the temporal coincidence with the maximum crawler movement (table 4), suggests that earwigs could be good candidates as biocontrol agents of WAA in Mediterranean areas. The capacity of earwigs to control WAA populations (Stap *et al.*, 1987; Mueller *et al.*, 1988; Nicholas *et al.*, 2005; Helsen *et al.*, 2007) and their promotion through the use of additional shelters in orchards (Solomon *et al.*, 1999; Gobin *et al.*, 2006; Logan *et al.*, 2011) has been reported. Moreover, Noppert (1987) and Phillips (1981) even estimated that a minimum of seven earwigs per tree was necessary to control WAA in apple orchards from Northern Europe.

**Fig 2.**
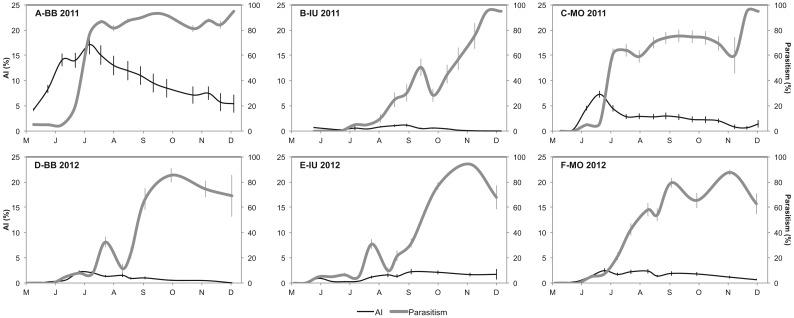
Woolly apple aphid aerial infestation (AI) and parasitism (mean±SEM) for each orchard and year.

Individuals of the WAA parasitoid *A. mali* were detected on the tapes from March to December, but parasitism on the canopy was recorded mainly from July to December (table 4 and fig 2). These observations reinforce the importance of promoting earwigs early in the season to maintain low levels of AI until the levels of parasitism by *A. mali* take over from summer onwards.

**Table 4.** Crawlers (up, down and canopy), *A. mali* and predators (earwigs, spiders and velvet mites) trapped on the glue tapes and the AI (mean monthly percentage of the total year data from all the orchards in 2011–2012).

Up	0	0	1	4	27	32	13	11	5	4	2	1
Down	0	0	1	3	21	34	16	16	3	5	1	0
Canopy	Not evaluated				24	10	29	30	3	3	1	0
Al	Not evaluated				6	19	20	22	12	6	10	5
Parasitism	Not evaluated				5	7	49	58	74	73	86	81
*A. mali*	0	0	12	5	2	9	16	20	9	18	8	1
Earwigs	1	0	2	9	34	31	11	2	1	1	3	5
Spiders	4	3	9	7	19	12	12	7	4	8	6	9
Velvet mites	1	3	10	6	1	1	10	16	11	24	12	5
		<5%				5% to <25%				> 25%		

Higher presence is shown by darker cells. Note that parasitism is represented by the mean recorded parasitism (%) for each month of all the years and of all three orchards.

Other WAA predators such as spiders and velvet mites were trapped from March to December (table 4), and due to this extended presence they could be considered candidates as predators of crawlers. Spiders have been reported as important predators of green apple aphid (*Aphis pomi* L., Hemiptera: Aphididae) and rosy apple aphid in orchards (Wyss *et al.*, 1995; Boreau de Roince *et al.*, 2013), while velvet mites are barely mentioned as predators of aphids (Helyer *et al.*, 2003; Marko *et al.*, 2008; Sundic & Pajovic, 2012).

The presence of spiders along the season and the temporal succession of earwigs and *A. mali*, suggests that biological control of WAA can be enhanced in orchards if these natural enemies are promoted or at least not disrupted.

### A multilateral approach to the role of biotic and climatic variables on the ecology of WAA

**Fig 3.**
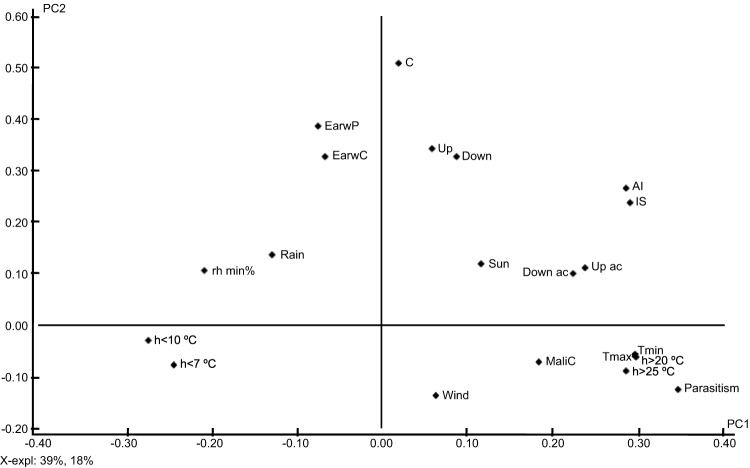
Variable loadings represented in the plane defined by the first two principal components. Variables are: the weekly number of aphids captured on the bands (Up, Down and Canopy (C)), the accumulated number of aphids captured each week (Up ac and Down ac), the percentage of aerial infestation (AI), the percentage of infested shoots (IS), the mean values of the classes of parasitism, the number of earwigs and *A. mali* individuals captured on the bands (EarwC and MaliC, respectively), and the number of earwigs present in shelters (EarwP), climatic variables such as maximum temperature (Tmax, °C), minimum temperature (Tmin, °C), number of hours above or below temperature thresholds (h>20 °C, >25 °C; h<10 °C and <7 °C), minimum relative humidity (rh min%), solar radiation (Sun, W m^−2^), rainfall (Rain, mm) and wind speed (Wind, m s^−1^).

Data from April to September, when WAA population dynamics mainly occurred, were used to construct a PCA. Although the complexity of the data determined nine principal components (PCs) to explain 90% of the variance, the first two PCs were able to explained 57% (39% PC1 and 18% PC2) of the overall variance (fig 3). The most important variables for the definition of the first PC were minimum and maximum temperatures (Tmin, Tmax) and the number of hours above or below several temperature thresholds (h<10 °C, <7 °C, >20 °C, >25 °C) (fig 3), suggesting that these climatic variables may have an important contribution to the WAA ecology. By the use of the diagram of scores, we observed that the variables defined in the direction of maximum information of the data (first PC) were clearly related to the week number of the year (data not shown). The second PC was determined by weekly crawler movement, such as that through the canopy (C), and upward (Up) and downward (Down) displacement, and by the presence of earwigs either on the glue tapes (EarwC) or in the shelters (EarwP) (fig 3). Therefore, as mentioned before, earwigs may decrease WAA number of crawlers moving up, down and through the canopy. The percentage of IS and the percentage of the shoot length occupied by WAA (AI) were highly correlated, and both variables were important in the definition of the first and the second PCs (fig 3). Therefore, as both variables are highly correlated, IS can be used instead of AI to evaluate the level of WAA infestation, as it is much easier to obtain.

The crawler movement through the canopy (C) did not have a close relationship with the AI or IS, nor with the crawler upward (Up) and downward (Down) movement (fig 3). These observations are consistent with the hypothesis mentioned above, that there is not a clear relationship between canopy and root colonies. Peak captures of crawlers moving through the canopy (C) were observed in the warmer months of the year (fig 1); however, with the multilateral approach we cannot confirm a clear correlation of canopy movement with the temperatures, and it may be more related to other variables not yet detected. The variables Up ac and Down ac had a high negative correlation with the number of hours below 10 °C (h<10) and 7 °C (h<7) (fig 3), suggesting that crawlers moving up and down the trunk will be more important when temperatures are higher than 10 °C. Hoyt & Madsen (1960) also reported that temperatures below 10 °C inhibited crawler movement in laboratory conditions.

We found no clear relation between rainfall (Rain) and relative humidity (rh min %) with crawler movement (Up, Down, C) and canopy infestations (AI or IS) (fig 3). A negative influence of rainfall on crawler migration was observed by Hoyt & Madsen (1960), Bhardwaj *et al*. (1995), and Heunis & Pringle (2006). The lack of correlation that we observed may be explained because in the conditions of our study, maximum crawler captures were observed during the driest weeks of the year, when rainfall was rare, more similar to the conditions in which Beers *et al.* (2010) performed their study in Washington.

The strong correlation observed between EarwP and EarwC suggests that glue tapes are a practical and efficient means by which to estimate the presence of earwigs in the orchard, without the need for special shelters. The number of *A. mali* trapped on the tapes (MaliC) appeared to be negatively correlated with rainfall (Rain) and not correlated with parasitism. Many *A. mali* were found on the tapes at the beginning of spring. This observation could be attributed to these insects emerging from overwintering mummies. The positive correlation found between parasitism and temperatures above 20 °C (fig 3), is consistent with the high rates of parasitism that we recorded from July onwards (fig 2) and with the observations made by Monteiro *et al.* (2004) in the warmer climate of Brazil.

Spiders and velvet mites were ruled out as main variables of the PCA as they had a null contribution to the overall explained variance. Wind and solar radiation (Sun) did not make an important contribution to the overall variance as well (fig 3), the study area was not especially windy, and the solar radiation was not limiting in any of the orchards. In contrast, Hoyt & Madsen (1960) suggested the relevance of solar radiation on daily crawler migration, as they observed the greatest movement in late afternoon and very little during darkness; however, in our analysis this daily dynamic was not observed as we recorded weekly captures.

**Fig 4.**
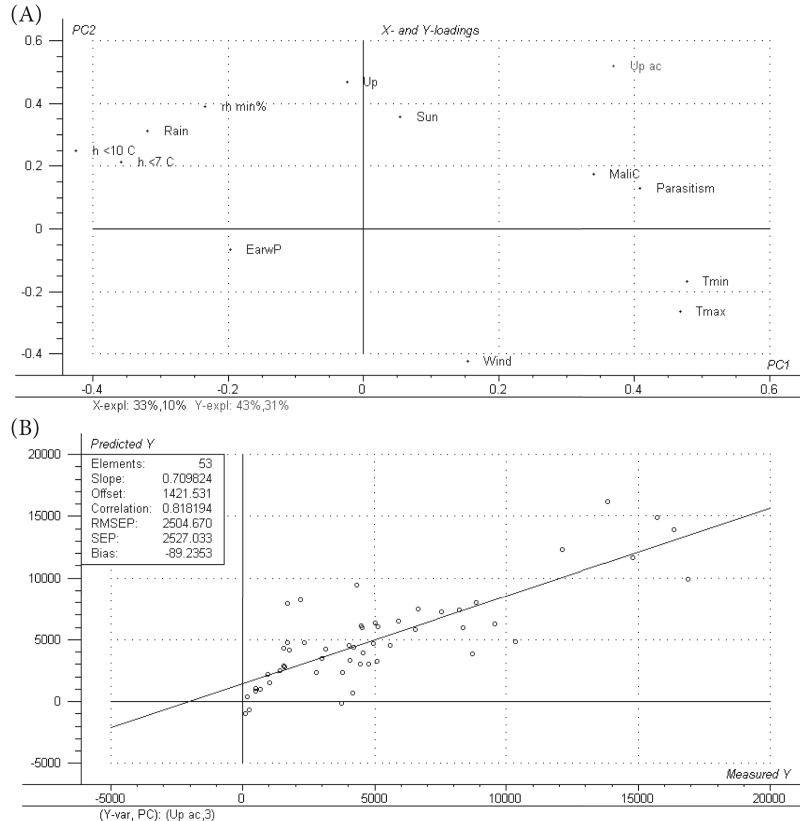
Up ac PLS-1: *X* and *Y* loadings represented in the plane defined by the two first PLS factors (A) and predicted versus measured diagram for the regression model of Up ac on the 12 variables analyzed (B). Variables are: the weekly number of aphids captured on the upper band (Up), the accumulated number of aphids captured each week on the upper band (Up ac), the mean values of the classes of parasitism, the number of *A. mali* individuals captured on the bands (MaliC), the number of earwigs present in shelters (EarwP), climatic variables such as maximum temperature (Tmax, °C), minimum temperature (Tmin, °C), number of hours below temperature thresholds (h<10 °C and <7 °C), minimum relative humidity (rh min%), solar radiation (Sun, W m^−2^), rainfall (Rain, mm) and wind speed (Wind, m s^−1^).

Regarding the PLS-1 to predict Up, the first two PLS factors explained 52% of the variance of the X-variables and only 26% of the information concerning the Up with a Root Mean Square Error of Prediction (RMSEP) value of 372.94 (data not shown) within a 0–2000 data rank. With these results, the model was considered not to be accurate enough to predict Up. On the other hand, in the PLS-1 method used to predict Up ac, 43% of the information contained in the X-variables explained 74% of the Y information (fig 4A). The latter model showed a coefficient of determination of 0.82 between predictions and reference values, and a RMSEP value of 2504.67 (data rank 0–20 000) to predict the Up ac between April and October. These values suggest that a reliable model can be constructed to predict the accumulated number of crawlers and that variables in addition to Up, such as MaliC, Parasitism, EarwP, Wind, Tmin, Tmax, Sun, rh min, Rain, h<10 and <7 have to be taken into account (fig 4). To reduce the unexplained variance (26%) additional variables not evaluated in this study that could have a direct effect on WAA or through an effect on natural enemies should also be included in the model.

**Fig 5.**
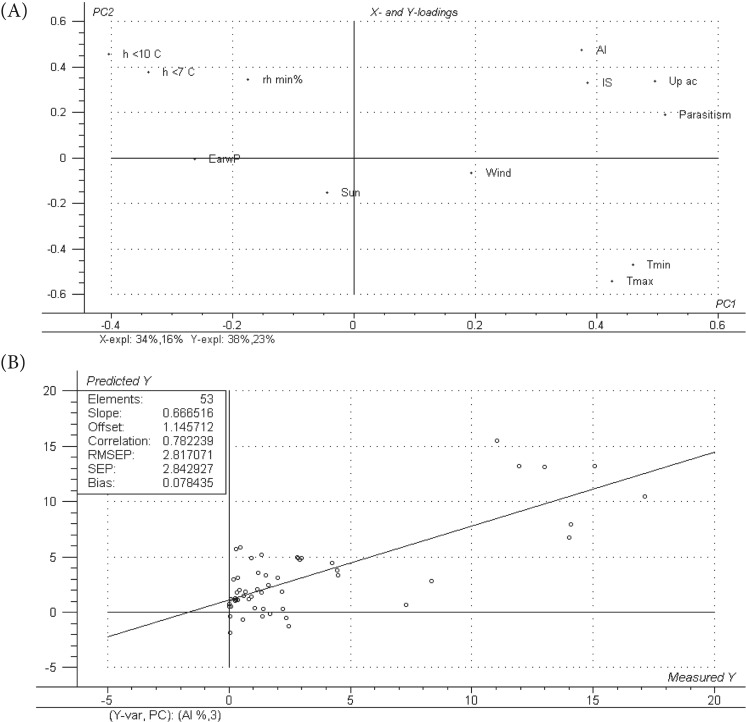
AI and IS PLS-2: *X* and *Y* loadings represented in the plane defined by the two first PLS-factors (A) and predicted vs. measured diagram for the regression model of AI-IS on the 10 variables analyzed (B). Variables are: the accumulated number of aphids captured on the upper band each week (Up ac), the percentage of aerial infestation (AI), the percentage of infested shoots (IS), the mean values of the classes of parasitism, the number of earwigs present in shelters (EarwP), climatic variables such as maximum temperature (Tmax, °C), minimum temperature (Tmin, °C), number of hours below temperature thresholds (h<10 °C and <7 °C), minimum relative humidity (rh min%), solar radiation (Sun, W m^−2^), rainfall (Rain, mm) and wind speed (Wind, m s^−1^).

In the PLS-2 procedure used to jointly analyze AI and IS, the first two PLS factors explained 51% of the variance of the *X*-variables and 61% of the *Y* information (fig 5A). The model obtained had a coefficient of determination of 0.78 between predictions and reference values and an RMSEP value of 2.82 (fig 5B) within a data rank of 0–20. These results were similar to those obtained from the PLS-1 to predict Up ac. The same considerations regarding the way to improve this model would also be suitable in this case. The contribution of earwigs appears again in both PLS-1 and PLS-2 models, with a negative correlation with the number of crawlers cumulated over the year and the canopy infestations (fig 4 and fig 5A).

For WAA, only linear models based on temperature (Asante *et al.*, 1991) or on developmental times (Bodenheimer, 1947; Evenhuis, 1958; Bonnemaison, 1965) have been reported. To our knowledge, this is the first approach aimed at modeling canopy infestations and crawler movement of WAA.

## Conclusions

The aim of this study was to provide knowledge to improve WAA management in Mediterranean areas. We conclude that both canopy and root colonies are the source of reinfestations in Mediterranean areas, as crawlers migrated upward and downward throughout the year and winter temperatures did not kill the aerial colonies. Therefore, measures of control must be addressed as well on roots as on the canopy.

Earwigs were found to reduce the survival of overwintering canopy colonies up to June. Predation of such colonies by earwigs in early spring is important to maintain them under low levels, allowing their later control by the parasitoid from summer to fall. Therefore, it is important to promote or at least not to disrupt neither earwigs nor *A. mali* in order to enhance natural control of WAA.

To improve the accuracy of the models in the prediction of canopy infestations, other variables that could affect WAA and/or natural enemies must be included. Further research is needed to determine an infestation threshold in spring to evaluate whether the natural control would be enough or if additional measures must be applied.
